# Inflammatory patterns in patients with chronic hepatitis C

**DOI:** 10.1186/1471-2334-13-S1-O32

**Published:** 2013-12-16

**Authors:** Daniela Adriana Ion, Mihaela A Rădulescu, Victoria Aramă, Raluca I Mihăilescu, Cătălin Tilişcan, Violeta Molagic, Cristina Popescu, Anca-Ruxandra Negru, Viorica Poghirc, Daniela Adriana Ion, Adrian Streinu-Cercel, Sorin Ștefan Aramă

**Affiliations:** 1 National Institute for Infectious Diseases “Prof. Dr. Matei Balş”, Bucharest, Romania; 2 Carol Davila University of Medicine and Pharmacy, Bucharest, Romania

## Background

In patients with chronic hepatitis C (CHC), liver fibrosis is a complex process, not completely understood, resulting from the activation of hepatic stellate cells and a pro-inflammatory status.

We aimed to evaluate the inflammation patterns in patients with CHC and the correlations between the inflammatory biomarkers and liver fibrosis.

## Methods

We performed a cross-sectional study on patients with CHC, in a tertiary-care hospital in November 2011 – April 2012. We staged liver fibrosis using FibroMax (BioPredictive) and we used CobasTaqman (Roche) for HCV-RNA quantification. Patients were also evaluated by ALT, platelets, erythrocyte sedimentation rate (ESR), fibrinogen, C-reactive protein (CRP) and tumor necrosis factor alpha (TNFa) plasma levels. For TNFa quantification we used Kamiya Biomedical Company ELISA kits.

## Results

We enrolled 76 patients with CHC (sex ratio M/F 0.6/1, median age 51 [44-58] years). According to the FibroMax evaluation, most of the patients had F0 fibrosis (25%, n=19), F2 fibrosis (42%, n=32) and F4 fibrosis (16%, n=12) respectively. The median HCV-RNA was 5 [5-5.75] log_10_. History of previous HCV-therapy with pegIFN and ribavirin was recorded in 75% (n=57) of patients and half of them had a sustained virological response.

The inflammatory biomarkers displayed the following patterns: median ESR 12 [8-22.5] mm/h, median fibrinogen 295 [254-343] mg/dL, median CRP 0.55 [0.2-1.5] mg/L, median TNF alpha 6.38 [4.75-9.33] pg/mL.

Comparing the patients without fibrosis (F0) with the patients with fibrosis (F1-F4), we found a significant difference between TNFa values (patients with F0 - median TNFa 5.5 [4.0-7.1] pg/mL; patients with F1-F4 – median TNFa 7.3 [5.0-10.6]; p<0.001). In patients with fibrosis, we found a moderate correlation between TNFa values and fibrosis score, according to the FibroMax score (rho=0.4, p=0.002) and between TNFa values and ALT (rho=0.43, p=0.001).

## Conclusion

In patients with CHC, liver fibrosis is correlated with the levels of TNFa, as a biomarker of systemic inflammation.

